# Placental Microtransfusions Associated with Increased HIV Transmission from Mother to Child

**DOI:** 10.1371/journal.pmed.0030026

**Published:** 2005-11-22

Mother-to-child transmission (MTCT) is the predominant way that children become infected with HIV. MTCT can occur prenatally during pregnancy, perinatally during labor and delivery, and postnatally through breastfeeding. In the absence of specific interventions, approximately one-third of children born to mothers who are HIV-positive become infected themselves. Specific interventions, including prenatal HIV counseling and testing, antiretroviral prophylaxis, elective cesarean delivery, and avoidance of breastfeeding, have reduced MTCT to less than 2% in developed countries. In many less-developed countries, however, these interventions are not readily available, and even effective, shorter, and cheaper antiretroviral prophylaxis strategies have not yet been widely implemented or accepted. As a result, MTCT rates in lower-income countries remain high, accounting for an estimated 2,000 new pediatric HIV infections per day.

An estimated one-half of the infant infections occur during labor and delivery, but the exact mechanisms remain poorly understood. Two possible ways of transmission have been proposed: direct contact of infant mucosa with HIV-infected maternal body fluids, and placental microtransfusions after breakdown of the maternal–fetal barrier through contractions at the beginning of labor. However, the evidence implicating either transmission route has been inconclusive.

To further examine a possible connection between placental microtransfusions and HIV MTCT, Jesse Kwiek and colleagues have taken advantage of a recently developed assay that provides a surrogate measure for placental microtransfusions, based on the amount of placental alkaline phosphatase (PLAP) in umbilical cord blood. PLAP is a large maternal enzyme that cannot cross the intact placental barrier. Infants produce very low levels of PLAP, and amounts found in umbilical cord blood are thought to result from leakage of maternal protein into the fetal circulation, caused by placental microtransfusions.

The researchers measured PLAP activity in umbilical cord blood as an indicator of maternal–fetal microtransfusions, and related this to risk of MTCT in a case-cohort study of mothers who were HIV-positive in Malawi. In the study, 149 women randomly selected from a larger cohort of pregnant women infected with HIV served as a reference group for 36 cases of prenatal MTCT and 43 cases of perinatal MTCT. The researchers saw no correlation between PLAP levels and prenatal MTCT. However, among the cases of perinatal transmission in women who had vaginal deliveries, elevated PLAP levels were associated with higher transmission risks. The connection was also seen after adjusting for some potential confounding factors such as HIV viral RNA load and chorioamnionitis.

While these are preliminary results and many questions remain, the reviewers felt that the design of the study enabled an efficient first test of the maternal–fetal microtransfusion hypothesis of MCTC. As such, it should encourage other researchers to look at this issue in their datasets. If a connection between microtransfusions and transmission is confirmed, it might help to improve the timing of short-term prophylaxis regimens and possibly lead to the development of new strategies for preventing MTCT of HIV.

